# *Fusarium verticillioides* of maize plant: Potentials of propitious phytomicrobiome as biocontrol agents

**DOI:** 10.3389/ffunb.2023.1095765

**Published:** 2023-02-07

**Authors:** Oluwadara Pelumi Omotayo, Olubukola Oluranti Babalola

**Affiliations:** Food Security and Safety Focus Area, Faculty of Natural and Agricultural Science, North-West University, Mmabatho, South Africa

**Keywords:** biocontrol agents, *Fusarium verticillioides*, maize pathogen, maize rhizosphere, phytomicrobiome, fumonisin, mycotoxin

## Abstract

Disease outbreaks have been recorded due to exposure to *Fusarium verticillioides* and fumonisin, a mycotoxin produced by this fungus. *F. verticillioides *is a fungal pathogen of maize that causes infections, such as wilting and rotting, while contact with its fumonisin derivative manifests in the form of mild to severe illnesses in humans and animals. Maize infection by *F. verticillioides* causes loss or reduction in expected crop yield, thereby influencing households and nations’ economies. While several efforts have been made to control the pathogenic fungus and its occurrence in the environment, it remains a challenge in agriculture, particularly in maize production. Several microorganisms which are plant-associated, especially those associated with the rhizosphere niche have been noted to possess antagonistic effects against *F. verticillioides*. They can inhibit the pathogen and tackle its debilitating effects on plants. Hence this study reviews the use of rhizosphere-associated biocontrol agents, such as *Bacillus *spp.*, Pseudomonas, Enterobacter*, and *Microbacterium oleivorans* which forms part of the phytomicrobiome in other to prevent and control this toxicogenic fungus. These microorganisms were found to not only be effective in controlling its occurrence on maize plants but are environmentally safe and promote crop yield.

## 1 Introduction

*Fusarium verticillioides *remains a major threatening pathogen in agriculture, particularly in maize production. This toxicogenic fungus possesses the ability to survive under extreme conditions, such as high temperatures (Czembor et al., 2015), and produces airborne spores, which explains its wide occurrence in the environment. It is a soil-borne pathogen, a part of the microbial community in the maize rhizosphere, which can contaminate the growing seed and roots of the maize plant, after which it moves up the plant itself (1).

It infects maize plants through contaminated seeds and contact of its contagious spores with the maize silk, while the invasion of maize kernels happens through cracks in the pericarp and the pedicel (Headrick and Pataky, 1991; Munkvold and Carlton, 1997; Munkvold et al., 1997; Oren et al., 2003; Ncube et al., 2017). The fungus can migrate to the soil through infected stalks or seeds (Oren et al., 2003; Ncube et al., 2017), with infection of maize by this pathogen being enhanced by late-season rainfall and the physiological state of the silk after pollination (Ncube et al., 2017).

*F. verticillioides, *which* *belongs to the fungi kingdom and Nectriaceae family, evolved approximately 7.3 million years ago following the divergence of the African and Asian clades of the *Fusarium fujikuroi* species complex (1). The fungus has been classified as part of the African clade, which produces a wide range of secondary metabolites (1). *F. verticillioides* is mainly associated with maize (1), where it colonizes the root, stem, and ears, resulting in diseases such as *Fusarium* ear rot, stalk rot, or even loss of the plant. According to Leslie et al. (1990), the fungus exists in crop residues in the soil, thus accounting for its occurrence in planted maize. It is a major pathogen and can be present in this crop without the plant showing any symptoms, its occurrence poses a severe risk due to its production of fumonisin (a kind of mycotoxin), which occurs frequently, and sometimes in high concentration. It also poses a major threat to the health of consumers (humans and animals), as it has been associated with illnesses such as esophageal cancer in humans (Shephard, 2011), and leukoencephalomalacia in equines and rabbits (Marasas, 2001; Giannitti et al., 2011).

The international agency for cancer research classified fumonisin as a possible human carcinogen (Humans IWGotEoCRt Cancer IAfRo, 2002), making the prevention of its occurrence in food advisable, this being more achievable (Omotayo et al., 2019) than its elimination. Several strategies (mechanical, physical, chemical) have been used to control the occurrence of various fungi and their derivatives in plants and foods, including the use of chemical disinfectants, such as sodium hypochlorite and chlorine dioxide. According to Chepkirui (2013), preventing fungi contamination in the field is regarded as a more effective method than post-harvest eradication. Several biological agents have been studied and proposed, being regarded as more promising and safer for plants, soil microorganisms, and the environment (Babalola and Glick, 2012).

The occurrence of this fungus in the maize rhizosphere ecosystem and the ubiquity of its fumonisin derivative, as well as their impact on the environment and plants, is hereby reviewed. This review also emphasizes the intensification of biological control agents for addressing *F. verticilloides’* occurrence before maize cultivation and during growth instead of chemical pesticides. The introduction of these in the field can serve as a control strategy, which can result in maximum yield and production of quality maize with little or no toxins (1). It will also reduce the airborne spores from the environment and hence minimize the risk of contamination (Colmenarez et al., 2018).

## 2 Method of literature search

**Figure 1:**
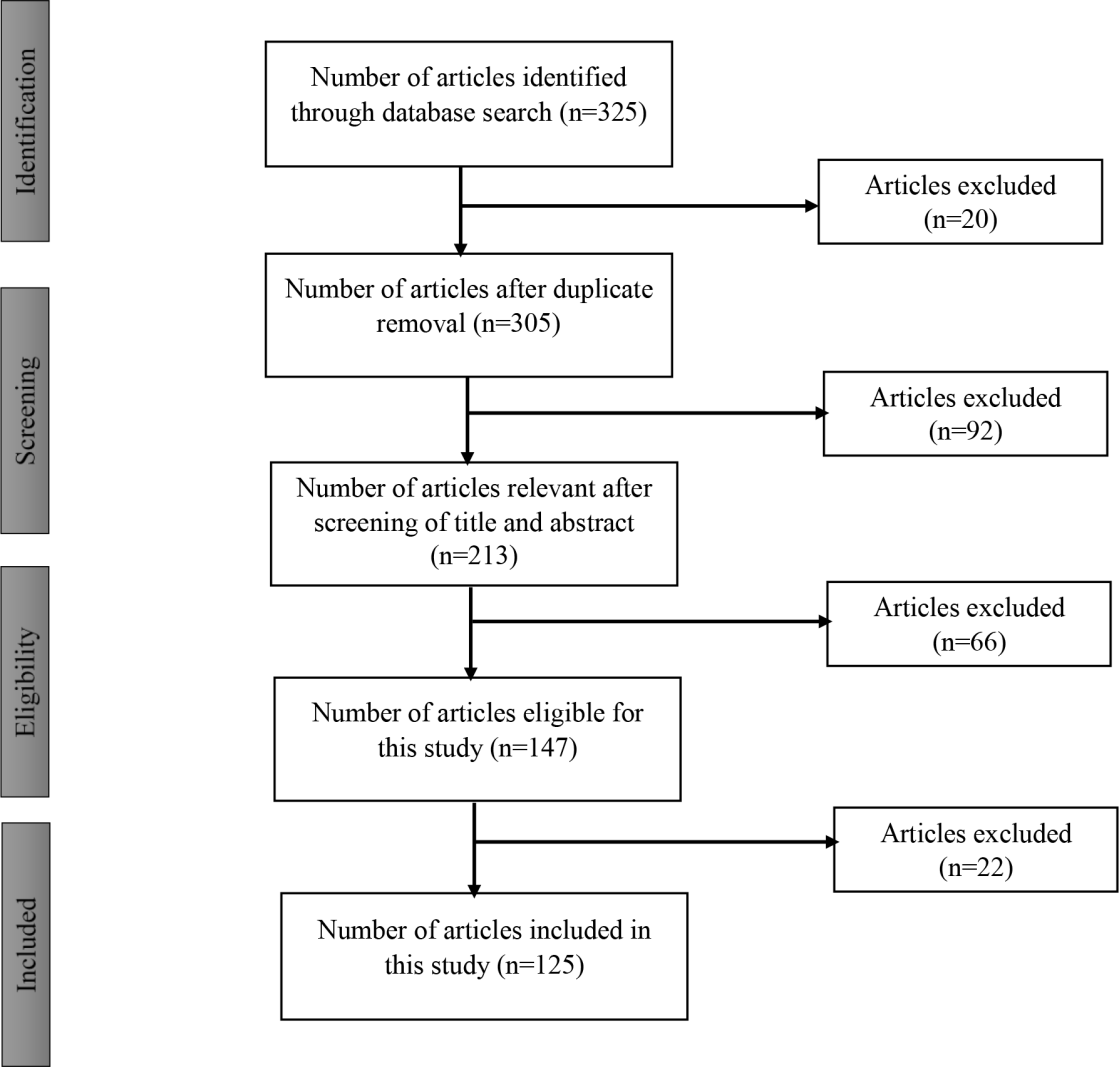
Flow diagram for selection of articles.

A literature search on *Fusarium verticillioides *and fumonisin, their effects, as well as biological agents capable of inhibiting these were conducted on materials published from 1968 to 2022 through scholarly search engines such as Scopus, PubMed, Web of Science, Google Scholar, and Science Direct. The keywords of this article (Biocontrol agents, Environmental pollution, *Fusarium verticillioides, *Maize pathogen, Maize rhizosphere, and Fumonisin) were majorly used for the search, with 325 articles being downloaded for review. A purposive selection of the articles was carried out yielding 125 articles (38% of the total downloaded articles) that were included in this review (**Figure 1**).

## 3 The impacts of *F. verticillioides* on plant, animal, and human health

*F. verticillioides* is a mycotoxigenic pathogen of maize that belongs to the fungi kingdom and the largest phylum of fungi, known as Ascomycota. The spores produced by this fungus contaminates the environment and poses a serious threat to human and animals. It was identified to be responsible for the Equine leukoencephalomalacia (ELEM) outbreak in South Africa in 1970 (Marasas et al., 1976; Omotayo et al., 2022). The pathogen was later found to be a causing agent of liver toxicity in rats and pulmonary edema in pigs (Kriek et al., 1981). In the United States of America (2), *F. verticilloides* was recorded to cause an outbreak of ELEM in 1989 (Marasas, 2001). The occurrence of this fungus in consumed maize has also been linked to the induction of proliferative hepatocyte lesions and tumors of the bile ducts in horses (Thiel et al., 1991; Alamu et al., 2016). In Argentina, an ELEM outbreak was recorded in 1945, followed by China in 1957, Egypt in 1968, South Africa in 1970, and Australia in 1999 after the consumption of contaminated maize. **Table 1** shows other effects and consequences of *F. verticillioides* and fumonisin on plants and animals.

**Table 1:** Other effects of *F. verticillioides *and Fumonisin* *on the plant, animal, and human health.

Pathogens/Mycotoxin	Effects	References
*Fusarium verticillioides*	Causes notable decrease in chlorophyll concentration in maize plants Results in plant water loss Causes root rot, ear rot, stalk rot and seedling blight in maize Results in stunted growth and chlorosis in plants Causes cutaneous lesions and mycotoxicosis in humans Can cause septic arthritis and endophthalmitis in humans Can result in Osteomyelitis and intracranial abscess in humans	(Sanchez et al., 1983; Munkvold and Carlton, 1997; Ncube et al., 2017; Baghbani et al., 2019) (Munkvold et al., 1997) (Headrick and Pataky, 1991; Stagnati et al., 2020) (O’Donnell et al., 2013) (Gupta et al., 2000; Hirata et al., 2001; Nucci and Anaissie, 2002; Schoeman et al., 2018) (Leslie et al., 1990; Hirata et al., 2001)
Fumonisin	Causes loss of plant yield Causes human foetal death Contamination with fumonisin causes catarrhal enteritis in human Lead to neural tube defects and cancer in humans Causes liver necrosis and cytotoxicity in humans Causes intestinal villous atrophy and absorption disorder Causes lymphoid depletion in piglets Causes histological lesions in the heart and kidney of piglets and rickets in chicks Causes oesophageal cancer in humans Causes nephrotoxicity and carcinogenesis in humans Causes hepatotoxicity and carcinogenesis in humans Causes pulmonary oedema in humans	(Shephard, 2011; Lorenzini and Zapparoli, 2015; Kamle et al., 2019) (Marasas, 2001) (Giannitti et al., 2011) (Humans IWGotEoCRt Cancer IAfRo, 2002; Marasas et al., 2004; Peterson et al., 2014; Omotayo et al., 2019) (Myburg et al., 2002; Chepkirui, 2013; Samir et al., 2018) (Babalola and Glick, 2012) (Taranu et al., 2005; Babalola and Glick, 2012; Chain EPoCitF et al., 2018; Terciolo et al., 2019) (Ledoux et al., 1992; Fazekas et al., 1998; van Lenteren et al., 2018) (Marasas et al., 1976; Marnewick et al., 2009; Colmenarez et al., 2018) (Demir et al., 2010; Omotayo et al., 2022) (Kriek et al., 1981; Voss et al., 2001) (Thiel et al., 1991; Alamu et al., 2016)

## 4 Infection routes for *F. verticillioides* and suitable environmental conditions for fumonisin production

In the field, *F. verticillioides *infects the maize plant, causing the production of fumonisin in the maize crop (Fandohan et al., 2006; Matić et al., 2013; Burger et al., 2013), which is often more concentrated in the embryo and pericarp of the maize kernel (Kimanya et al., 2008; Burger et al., 2013; Czembor et al., 2015). The growth of this fungus and fumonisin are favored by various factors such as high temperature (30-35°C) and the composition of kernel endosperm (Chulze, 2010; Picot et al., 2011; Alberts et al., 2016). These factors result in the fungi occurring in temperate and Mediterranean climate regions as the weather conditions are favorable to toxin accumulation in maize plants during their growth and development, particularly at the flowering stage (Kamle et al., 2019). Apart from higher temperatures, growing hybrids outside their adaptation areas, drought stress, and insect herbivory can also lead to infection by this pathogen (Betz et al., 2000; Alberts et al., 2016; Li et al., 2017). When comparing the fumonisin content in maize from different zones in Nigeria, Fandohan, et al. (Fandohan et al., 2005) observed that maize from regions with higher humidity levels has more fumonisin concentration than others. This study agrees with the findings of Mupunga (2013), on the correlation between fumonisin and high humidity.

The fungi* *gain access to the maize kernels commonly through wounds caused by insects such as the European corn borer, earthworms, and birds, and can also penetrate the maize through the silk by growing through the silk channel to the ear tip (Scarpino et al., 2015; Oldenburg et al., 2017). After harvest, some factors have been shown to contribute to fumonisin production in foods, these include high humidity, poor storage facilities, improper sorting, and unhygienic environments (Kamala et al., 2016; Moss and Frank, 1985). Furthermore, poor storage facilities prone to insect infestation and high moisture could lead to the production and contamination of foods by fumonisin. When sorting (which involves the removal of damaged, discolored, and molded grains) is not carefully done before storage, contamination with this pathogen might occur. It has been reported that insects like the European corn borer make a wound on the maize kernels which could create an avenue for infection by these fungi (Scarpino et al., 2015; Oldenburg et al., 2017) thereby causing fumonisin contamination.

### 4.1 Infection mechanisms of *F. Verticillioides*

**Figure 2:**
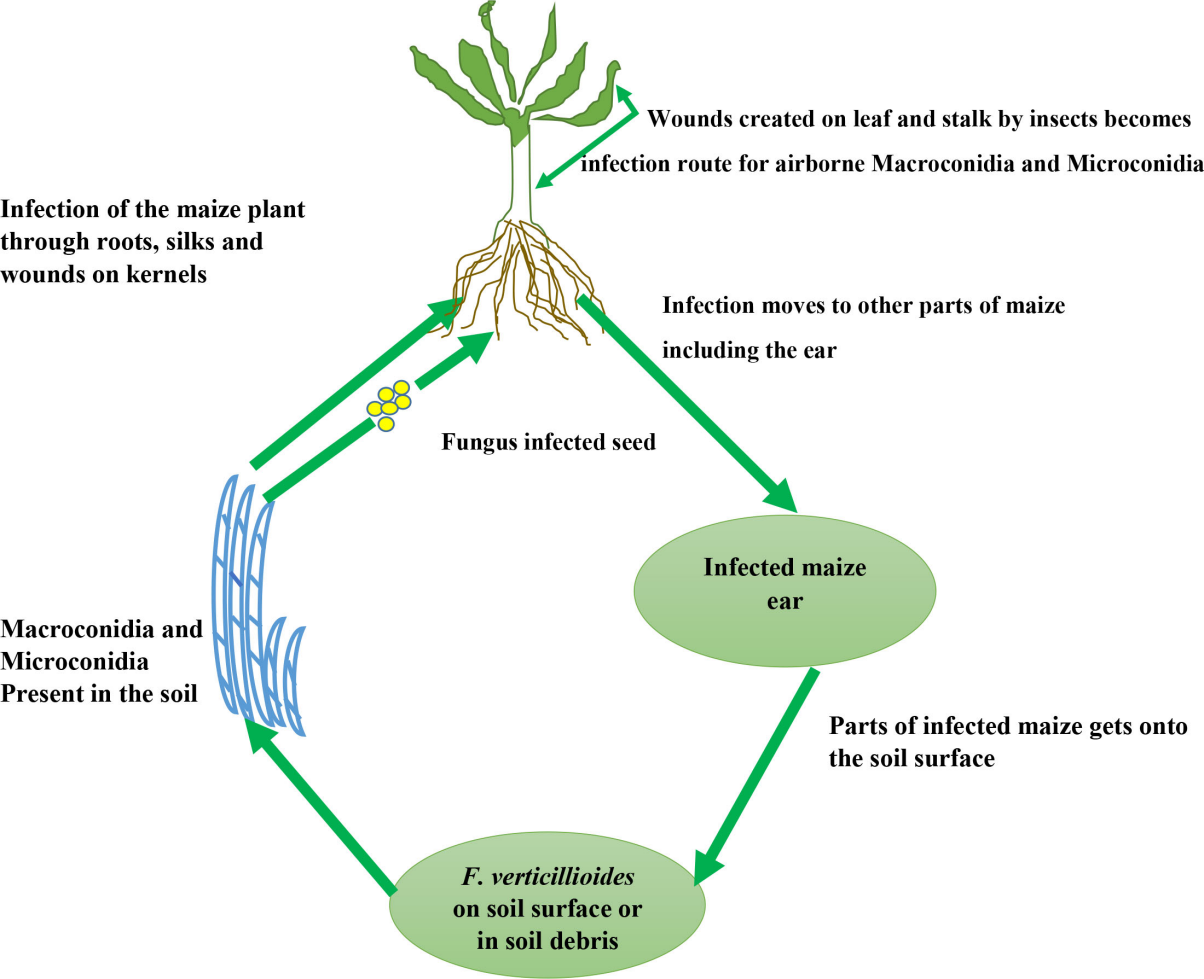
Infection cycle of *F. verticillioides* in maize.

Insects such as the European corn borer and western corn rootworm play a major role in *F. verticillioides’* infection and cycle as they feed on maize ears and stalk respectively (Munkvold and Carlton, 1997; Oldenburg et al., 2017). Wounds created on the stalk by the western corn borer become infection routes/sites for *F. verticillioides *stalk rot under dry conditions (**Figure 2**). Likewise, the egg deposited by the European corn borer moth on the leaves surface develops into a larva and thus feeds on leaf tissues and stalks thereby providing infection courts for the development of *F. verticillioides *stalk and ear rots (Blacutt et al., 2018). These larvae proliferate and deposits another set of eggs across the plant tissues (Sobek and Munkvold, 1999) which develops into Larva and creates more route for *F. verticillioides *through wounds created on stalks.

**Figure 3:**
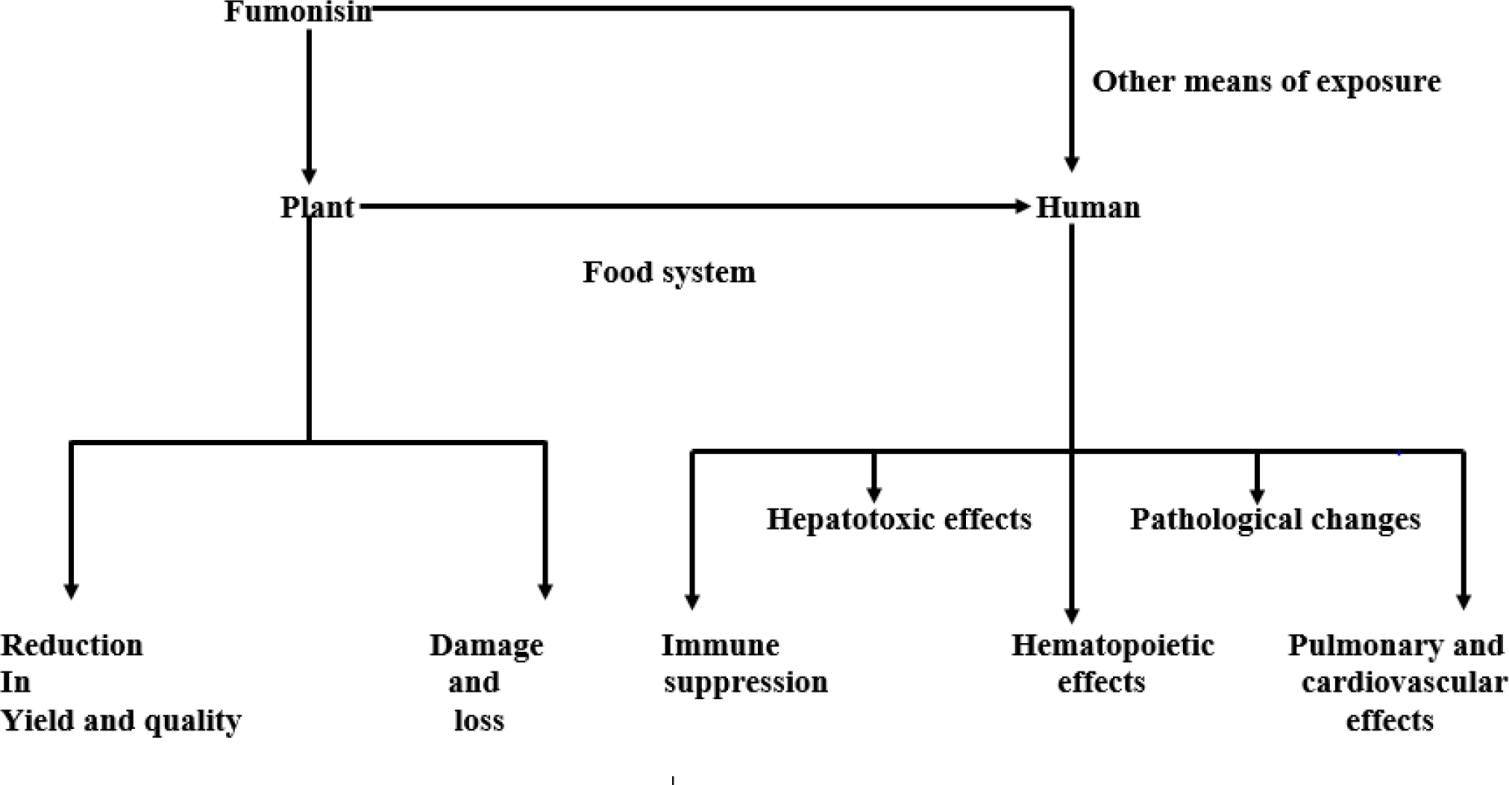
Effect of fumonisin on plant and human health.

Also, seeds planted in soils infected with *F. verticillioides *can become contaminated with the pathogen thus causing infection of the whole plant (Sobek and Munkvold, 1999) (**Figures 2**, **3**). According to Blacutt et al. (2018), and Oren et al., (1), under favorable conditions for the seedlings, the fungus grows in a hyphal manner and progressed along the root surface, sparsely penetrating the lateral roots and mesocotyl about 72 hours after planting into infested soils. Thereafter, it progresses into the mesocotyl tissue within seven days, and in about fourteen days, it grows into aerial tissues although in an asymptomatic manner and undetectable by fluorescence microscopy. According to Blacutt et al. (2018), at this stage, the mesocotyl cells accumulate undefined organelles and are filled with conidia (which is essential for the systemic movement of *F. verticillioides* within the plant). After 30 days, according to the study of Oren et al. (1), the fungal biomass increase greatly resulting into necrosis of the mesocotyl and main root.

From the study of Duncan and Howard (2010), microscopic and histologic characterization at the silking stage showed that once the fungus has gained access to the maize plant, it populates young developing kernels through the stylar canal resulting in starburst pattern which is commonly seen/observed in crops like maize. Other insects important in the infection of *F. verticillioides *are the adult thrip and beetle which causes wound on maize ears thus promoting *F. verticillioides* infection. Through existing openings such as cracks in roots, lateral roots, and root hairs *F. verticillioides *can penetrate (1). Leaf stomata or trichrome can also serve as a route of infection of maize by *F. verticillioides* hyphae (Nguyen et al., 2016).

### 4.2 Pathogenicity of *F. verticillioides*

*F. verticillioides *can be a facultative endophyte that can exist by depending on the maize plant for survival without harming the host (biotrophically), as well as by relying on the nutrients from decayed maize rubble or soil (saprotrophically) (Samsudin, 2015). As an endophyte, it can be vertically transmitted through maize seeds, thus initiating asymptomatic and systemic infection of the plant. During systemic infection, the fungal conidia are carried inside the seeds or the surface of the seeds and later develop inside the growing maize plant. The developed *F. verticillioides* thereafter moves from the roots to the stalk, cobs, and kernels. The veins of the maize root can also be infested by some conidia and also grow along the root veins after germination, while some will infiltrate the plant cells, attach to them and form hyphae in other to penetrate the neighboring cells (Samsudin, 2015).

As a pathogen, in maize, *F. verticillioides *causes severe symptomatic infections such as root rot, stalk rot, seedling blight, and ear rot (Bacon et al., 2008). According to Samsudin (2015), this fungus moves from the contaminated soil upwards to the maize stalks, thus resulting in stalk rot which is presented by the rotting of the lower stems, maize internodes, and roots. Moreover, it can survive through the winter as viable spores and becomes airborne or splashed by rainfall, thus causing infection of the silks during flowering. Diseases caused by *F. verticillioides *like ear rot in maize (which is the most damaging and common fungal-borne disease in maize worldwide) decrease quality, and yield and make the maize kernel vulnerable to fumonisin contamination (Khan et al., 2020). Consumption of maize polluted by this mycotoxin causes severe diseases in humans and animal (Blacutt et al., 2018).

## 5 Overview of *F. verticillioides* and fumonisin

The invasion and occurrence of *F. verticillioides* in maize have continued to create persistent setbacks in its production, with several approaches having been used to address its presence, one being chemicals. According to the literature, some chemicals including captan + thiabendazole and fludioxonil + metalaxyl - M have been used to control the pathogen, but the possible shortcomings and deleterious effects on humans, animals, and other beneficial microbes in the soil is a challenge to their use (Nicolopoulou-Stamati et al., 2016). For example, it has been reported that despite the ability of fungicides to suppress fungal activity and increase the growth and production of maize, it does not have a significant effect on mycotoxins reduction, including fumonisin, which is a derivative of these fungi but rather increases their levels (Bordini et al., 2015). Likewise, Tridemorph was found to increase the production of T-2 toxin, although it inhibited the growth of *Fusarium* *sporotrichioides* (1). Moreover, the use of Prochloraz and tebuconazole was observed to promote the biosynthesis of trichothecenes (Doohan et al., 1999), with Trans-2-hexenal being found to be effective in controlling *F. verticillioides*, but does not halt the production of fumonisin (Menniti et al., 2010). Fungicides are also limited in eradicating *F. verticillioides*, as they are not effective in the long term to have made a significant impact (Bordini et al., 2015).

The consequences of fumonisin (produced by these fungi) on agricultural production globally and human health cannot be overemphasized. The presence of this mycotoxin in plants results in damage and loss in crop and livestock production while its infiltration into the food system may cause significant havoc to the lives of humans and animals (Omotayo et al., 2019). The effect of fumonisin on plants is not only limited to damage and loss but also affects the yield and quality of the crop (Omotayo et al., 2019). This mycotoxin is capable of attacking crops at any developmental stage and causing various plant diseases before and after harvest (Iqbal et al., 2021). Upon invasion of the human body through the food system, fumonisin suppresses the immune system and reduces the human ability to resist environmental and microbial stress thus increasing its susceptibility to diverse diseases (Mita et al., 2022). While it is classified into three forms: FB1, FB2, and FB3, FB1 is the most abundant, prevalent, and toxic, and poses the greatest threat to plant, animal, and human health. It stresses the endoplasmic reticulum and causes cellular autophagy, cytotoxicity, oxidative stress, apoptosis, DNA damage, and consequently cell carcinogenesis. Following the alteration of several components of the human cells, tissues, and organs, it exerts hepatotoxic, hematopoietic, and other toxic effects on the human body and also induces pathological changes (Blacutt et al., 2018) (**Figure 2**).

The occurrence of *F. verticillioides* in maize is fast becoming a challenge that cannot be easily resolved, leading to problems in maize production. In the quest for lasting preventive measures and a remedy to the consequences of these fungi on maize production, the use of biological agents has been examined. It was found that some organisms are capable of limiting the activity of this pathogen (Khan et al., 2017), thereby limiting/reducing its negative effect (Bressan and Figueiredo, 2005). It was also noted that biological agents are friendly to the atmosphere (Thavaselvam and Vijayaraghavan, 2010) and are also target-specific, as they only attack the pathogen (Usta, 2013). The rhizosphere has been noted to host some of these organisms, which can be enhanced to control *F. verticillioides*, some of these include members of the genera *Bacillus*, *Enterobacter*, *and Pseudomonas*.

## 6 Mode of action of *F. verticillioides* and fumonisin

**Figure 4:**
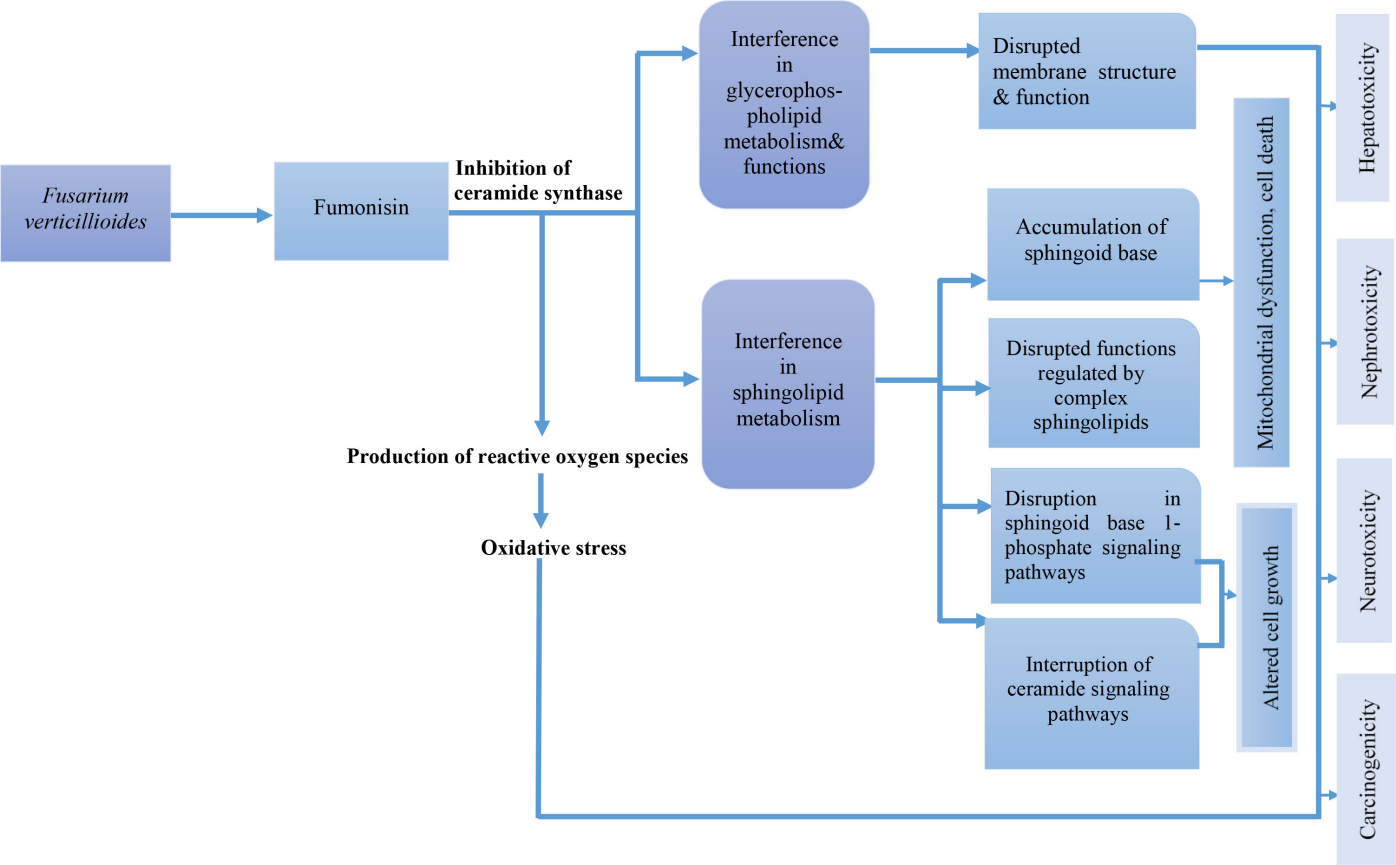
Mode of action of fumonisin.

The effects of biotic and abiotic factors such as plant genetics, high temperature, and low humidity results in the production of fumonisin from *F. verticillioides. *Fumonisins (FB1 and FB2)* *are structural analogs of sphingoid bases and they inhibit ceramide synthases (which acylate sphingoid bases) and sphingosine-sphinganine-transferases leading to the interference of the sphingolipid metabolism and accumulation of sphinganine and sphingosine in cells and tissues. Inhibition of ceramide synthases results in the production of reactive oxygen species which increases oxidative stress and causes the induction of lipid peroxidation and cell damage. Sphinganine and sphingosine-induced apoptosis, exert growth inhibitory effects and the accumulation of these two (sphingoid bases) is the primary cause of fumonisin toxicity (especially FB1). **Figure 4** summarizes the mode of action of fumonisin.

## 7 Role of microbial inoculants in the management and control of fumonisin

While the complete eradication of mycotoxins including fumonisin remains a challenge, biological measures can be taken to prevent their invasion, control their spread and manage their effects. This involves the introduction of microbes such as *Trichoderma harzianum* and *Ruminococcus flavefaciens* (1). *Trichoderma harzianum *is a fungus present in the soil and commonly used in foliar preparation for animals, thus leading to the presence of these microorganisms in the gut of ruminant animals. Studies have shown that this fungus not only resists *F. verticillioides* but also can be used in controlling fumonisin. The capability of ruminant animals such as cows to resist the effects of various mycotoxins consumed in forages, and their ability to modify and degrade toxic substances is evidence of the potential of this cellulolytic microorganism in mitigating the effect of fumonisin (Rodrigues, 2014). Hence, the application of this fungus as microbial inoculants to plants would help to modify and degrade fumonisin thus preventing its invasion of crops. From the study of Bakri (2021) which evaluated the effect of *T. harzianum* and other cellulolytic enzymes on divers mycotoxin, it was found that this fungus together with *Lactobacillus bulgaricus* greatly inhibited the production of fumonisin and other mycotoxins.

*Ruminococcus flavefaciens *is another gut microbe that can be isolated and optimized for the control of fumonisin. As shown in previous studies, this microorganism is capable of modifying mycotoxin’s toxicity by causing changes to its toxicokinetics (Aisha, 2012; Guerre, 2020). That is, this microbe can cause the chemical transformation of fumonisin through the enzymes present in its cell or through the enzymes excreted by the microorganism into the gut. This process leads to the hydrolysis of fumonisin and the formation of less toxic metabolites. The cell wall of this microbe also can bind fumonisin and alter/reduce its absorption (Guerre, 2020).

*Bacillus subtilis* CE1 is not only capable of inhibiting *F. verticillioides *but reduces fumonisin accumulation (Mita et al., 2022),. This bacteria which is commonly found in the maize rhizosphere is one of the numerous microbes which hinder the production and buildup of the mycotoxin derivative-fumonisin. Collection of fumonisin in plants is hazardous not only to the plant but the environment, however, *B. Subtilis* would help in preventing fumonisin contamination in plants, thus making them free from this hazardous mycotoxin.

## 8 General effects of *F. verticillioides* contamination

Apart from the effects of these disease-causing pathogens on the maize plant, it also impacts food production, the environment, and the economy negatively, thereby causing compromise of maize quality, food, and environmental pollution, which further results in disease outbreaks and health complications.

### 8.1 Food and environmental pollution

Cereals such as wheat, barley, millet, rye, oat, and most frequently maize are the most commonly infected food groups by *F. verticilloides*, although garlic, asparagus, and dried figs can be contaminated by its fumonisin derivative (Heperkan et al., 2012; Kamle et al., 2019). In the study conducted to identify the most prevalent *Fusarium *species in an environment, Czembor, (Czembor et al., 2015) found that *F. verticillioides* and *Fusarium temperatum *were the most prevalent in several maize samples. Likewise, Borah (Borah et al., 2016), identified *F. verticillioides* to be the most prevalent fungi causing an infection on maize cultivated in a region in Assam.

Due to the production and spread of airborne spores by *F. verticillioides*, exposed food products may become contaminated by contact, the inhalation of which can result in various illnesses in animals and humans. According to the study of Abiala and Odebode (2015), in which the presence of fungal spores in the atmosphere was investigated, spores of* F. verticilliodes* were among those collected from the air at various locations in Nigeria. The spores travel easily in the air when dispersed, thus polluting the atmosphere and posing various threats to plants and animals. An increase of these mycotoxin-producing fungal spores was observed during summer, which is in line with the study of Omotayo (Omotayo et al., 2019a),. These fungal spores are capable of contaminating food, as noted by Badar (Badar et al., 2012), who observed them on some vegetables and fruits in India.

### 8.2 Compromise of maize quality

Maize is a crop with high economic value and impacts globally, it helps to minimize food shortages and is capable of limiting hunger and starvation, and boosting the food security of developing nations (Adiaha, 2018). Maize production raises the standard of living and contributes to the increasing income of farmers and foreign exchange earnings (Omotayo et al., 2016; Adiaha, 2018; Omotayo et al., 2022). Millions of tons of maize are produced each year in South Africa, with approximately 12.5 million tons being produced in the 2018/2019 season, and 16.1 million tons in 2019/2020 (1).

In South Africa, maize has yielded over 15% of the gross value of all agricultural products and covered approximately 4% of the cultivated area (Stats, 2017). This widespread production of maize contributes to the income of farmers globally. However, *F. verticillioides* in maize reduces the quality of this crop (Ncube et al., 2011). This implies that inadequate control of this fungus can compromise the quality of maize unknowingly due to its asymptomatic outward appearance (Rai, 2011; Ribes et al., 2018). This does not only influence the income of farmers but households and maize-producing regions.

### 8.3 Disease outbreak and health complications

The presence of fumonisin in crops can infiltrate food and feeds during production (Kamle et al., 2019), causing various reactions, from allergy to several illnesses. In southern Africa, maize and its derivatives are a major source of food (Du Plessis, 2003), constituting a significant part of infants’ diets. The consumption of contaminated maize and its end products, therefore, affects the health of not only adults but also infants, leading to various complications (Omotayo et al., 2016; Gai et al., 2018; Omotayo et al., 2019b) and in severe cases, death.

## 9 The role of the phytomicrobiome in biocontrol

The microorganisms associated with plants (also known as the phytomicrobiome) interact with the host plant in various ways and perform crucial roles in agriculture (Leveau, 2015; Omotayo, 2022). *Bacillus amyloliquefaciens *and* Microbacterium oleovorans* are part of the microorganisms that dominate the phytomicrobiome and produce various kinds of antibiotics which serve the host plant in protection against the pathogen. From previous studies, it has been found that these plant-associated microorganisms which are majorly bacteria, fungi, and archaea can be manipulated and engaged as biocontrol agents against the notorious *F. verticillioides *(1).

## 10 Biocontrol agents

The introduction of materials with biological origin to regulate or eliminate target organisms has been considered effective and environmentally secure (English, 1970; Harper, 2001; Fendrihan and Pop, 2021). It involves the use of suitable and effective pathogens, predators, and competitors, which can be bacteria, fungi, viruses, and protozoans (English, 1970; Aremu et al., 2017). Examples of these are *Trichoderma *spp*.*, that is used against *Macrophomina phaseola* and *Rhizoctonia solani* to control charcoal rot and banded blight in maize, with *Bacillus* species being used against a wide array of pathogens, including *Xanthomonas oryzae* to control bacteria leaf blight in rice (Singh, 2014). *Streptomyces* isolates (designated as DAUFPE 11470 and DAUFPE 14632), that are isolated from the maize rhizosphere soil, have been proven to be effective in controlling *Stenocarpella maydis* in maize (Bressan and Figueiredo, 2005).

The above isolates suppress the development of *Penicillium* spp.,*Curvularia lunata*, *Drechsleramaydis*, *Aspergillus *spp., and other pathogenic fungi (Bressan and Figueiredo, 2005). However, the high specificity of these agents makes them environmentally benign, and their cost also has limited their use (Nations U, 1972). In the control of *F. verticillioides*, the following biological agents have been confirmed effective: *Bacillus amyloliquefaciens, Microbacterium oleovorans, Enterobacter *spp., *and Pseudomonas *spp.

### 10.1 Bacillus amyloliquefaciens and Microbacterium oleovorans

*Bacillus* species can produce resistant spores as well as antibiotics that are capable of inhibiting a wide range of plant pathogens by antibiosis (Cavaglieri et al., 2005; Adeniji et al., 2020). According to Khan (Khan et al., 2017), they resist numerous pathogens, including fungi, by producing certain metabolites that exert a toxic effect or create a systemic resistance in plants. The propensity of these microbes to withstand harsh environmental conditions, such as high temperatures, insufficient nutrients, inadequate water supply, and adverse pH (Shafi et al., 2017), enables them to thrive in several habitats, including soil and seawater (Hoch and Sonenshein, 1993). *Bacillus amyloliquefaciens* has been reported to be present in the maize rhizosphere due to its ability to survive under low oxygen concentrations (Nakano and Hulett, 1997), thus making it capable and effective as a biopesticide (Rosas-García, 2009) as well as a biocontrol agent against *F. verticilloides*.

Among these groups of organisms, *Bacillus amyloliquefaciens *impedes *F. verticilliodes* and other soil pathogens through the competitive exclusion principle when introduced into the soil before maize cultivation (Palma and Arteiro, 2007). It competes with this pathogen for nutrients while producing toxic compounds that are capable of inhibiting them (Palma and Arteiro, 2007). Pereira (Pereira et al., 2007), evaluated the ability of *Bacillus amyloliquefaciens and Microbacterium oleovorans *to* *reduce the occurrence of *F. verticillioides* in maize and showed that their application is effective. It was also found that *Bacillus amyloliquefaciens* can reduce the level of occurrence of this fungus in the maize rhizosphere (Pereira et al., 2007). Introducing it to the maize rhizosphere would bring about a substantial reduction in the spread of *F. verticilliodes* from the soil to the plant, hence reducing the fumonisin content in the maize.

Apart from the introduction of these biocontrol agents into the maize rhizosphere, seed coating with *Bacillus* *amyloliquefaciens* is also a technique that can be optimized to combat this pathogen (Chandra Nayaka et al., 2009). This will not only antagonize *F. verticilloides* in the rhizosphere but also repress it in the spermosphere, thus preventing its infiltration into the growing plant (Pereira et al., 2007). The observations of Siahmoshteh (Siahmoshteh et al., 2018), about the ability of *Bacillus subtilis* and *amyloliquefaciens* to produce antifungal substances that could subdue a wide range of pathogenic fungi associated with food, including maize, are also in line with the above findings.

Spraying maize ears at the flowering stage with *Microbacterium oleovorans *was effective in reducing the *F. verticillioides* count and its fumonisin B1 content in maize (Pereira et al., 2010). In the study by Pereira (Pereira et al., 2007), maize seeds were treated with *Microbacterium oleivorans* and then planted, after which it was found that treatment with the bacterium* *produced a significant reduction in the fungus counts at the root inner tissues of growing seedlings, and also reduced the quantity of Fumonisin (FB1 and FB2) significantly. According to Chulze (Chulze et al., 2015), *Microbacterium oleivorans *is effective in controlling this fungus and the accumulation of fumonisin in maize at the pre-harvest stage, these findings agree with that of Sartori (Sartori et al., 2012), who stated that its treatment with *Microbacterium oleivorans* significantly decreased its growth rate.

### 10.2 Enterobacter

Another species of bacteria that has been noted to be effective as a biocontrol agent against *F. verticillioides* is *Enterobacter hormaechei*, which belongs to the* *Enterobacteriaceae family, known previously as the Enteric group 75 (O'hara et al., 1989). The efficiency of rhizospheric *Enterobacter* in controlling *F. verticilliodes* especially when used with other bacteria such as *B. amyloliquefaciens* has been confirmed (Pereira et al., 2010). According to Pereira (Pereira et al., 2010), seed coating with *B. amyloliquefaciens* and *E. hormaechei* reduced significantly the level of fumonisin B1 in harvested maize*. *Moreover, Abiala and Odebode (2015), showed that rhizospheric Enterobacter species (OSR7 and IGGR11) are effective in suppressing *F. verticilliodes*. In their study, the potentials of rhizosphere Enterobacter species (OSR7 and IGGR11) on plant growth was explored, with the finding indicating that these bacterial species not only improved maize growth but also impeded the pathogenic activity of *F. verticillioides*, thereby leaving the crop with no symptom of the disease.

*Enterobacter cloacae* is another species noted to be effective in inhibiting *F*. verticillioides (Cavaglieri et al., 2005), *Enterobacter cloacae* together with *Microbacterium oleovorans, *were combined for treating maize seeds before cultivation. It was observed that the mixture inhibited the growth of *F. verticillioides*. According to Hinton and Bacon (1995), *Enterobacter cloacae* has been reported to be an excellent biocontrol agent against root colonization of this Fusarium species in maize.

### 10.3 Pseudomonas

These groups of microorganisms are known for their metabolic diversity and ability to occupy several habitats, being found in soil, water, and plants (Brock et al., 1994; Adeniji et al., 2020). *Pseudomonas* is a genus of Gram-negative Gammaproteobacteria, which belongs to the Pseudomonadaceae family (Euzéby, 2009). Nayaka et al. (Chandra Nayaka et al., 2009) observed that after spraying maize seeds with a pure culture and powder formulation of *P. fluorescens*, this biocontrol agent successfully inhibited the growth of *F. verticillioides* and suppressed the synthesis of fumonisin. Furthermore, antibiosis was observed by Cavaglieri et al. (Cavaglieri et al., 2005; Chandra Nayaka et al., 2009) when *Pseudomonas solanacearum* and *Bacillus subtilis* were used for maize seed treatment before planting, being able to antagonize *F. verticillioides* and diminish the production of fumonisin.

### 10.4 Burkholderia cepacia

Formerly known as *Pseudomonas cepacia*  (Laraya-Cuasay et al., 1977), this resides in the rhizosphere of many plants and is noted to produce active metabolites, which resist and inhibit many disease-causing fungi. Bevivino (Bevivino et al., 2005), confirmed the ability of this bacteria to inhibit the growth of *F. verticillioides*. According to Zeidan (Zeidan et al., 2019), the inhibitory activity of the thermostable metabolites of these bacteria makes them suitable biocontrol agents against toxigenic fungi. It was also discovered that the bacteria possess antifungal activity against mycotoxigenic and phytopathogenic fungi, their ability to inhibit *Aspergillus carbonarius* and *Penicillium verrucosum* being recorded. **Table 2** provides recommended strategies from the literature that can be adopted to effectively control *F. verticillioides* and its fumonisin derivative, and indicates how various organisms can be adopted in their control, such as colonizing the maize root with biological control agents, inoculation, seed priming/treatment as well as an in-planta assay.

**Table 2:** Other plant associated microorganisms with potentials for Fusarium verticillioides and fumonisin biocontrol.

Control strategies	Biocontrol agents	Effects of Biocontrol agents	References
Seed treatment/Biopriming/Inoculation/Spray	*Enterobacter cloacae* *Microbacterium oleovorans* *Pseudomonas solanacearum*	Inhibits root colonization	(Alamu et al., 2016)
	*Bacillus mojavensis* *Bacillus thuringiensis*	Suppresses and inhibits pathogenic activity	(Oren et al., 2003; Lorenzini and Zapparoli, 2015; Baghbani et al., 2019)
	*Trichoderma harzianum*	Controls fumonisin accumulation and *Fusarium verticillioides infection*	(Sanchez et al., 1983; Matić et al., 2013; Stagnati et al., 2020)
	*Enterobacter hormaechei*	Maximum reduction of ear rot disease, Inhibits growth rates of fungi, Reduces pathogen content in harvested grains	(Nucci and Anaissie, 2002)
	*Pseudomonas aeruginosa* *Bacillus subtillis* *Bacillus amyloliquefaciens*	Inhibits fungi growth rates and reduces fumonisin in maize	(Gupta et al., 2000)
	*Pseudomonas fluorescens*	Controls ear rot disease, reduces fumonisin accumulation in maize	(Peterson et al., 2014)
Root colonization, soil treatment/spray/inoculation	*Trichoderma harzianum*	Induces systemic resistance	(Sanchez et al., 1983)
	*Bacillus subtillis CEI* *Bacillus amyloliquefaciens*	Reduces rhizoplane and endorhizosphere colonization of these pathogens	(Kamle et al., 2019)
	*Kluyveromyces* sp. *L16*	Reduces fungi count in maize soil	(Chain EPoCitF et al., 2018)
	*Enterobacter* sp.	Suppresses pathogenic activity of *F. verticillioides*	(Marasas et al., 2004)
	*Bacillus* sp. *B25*	Decreases stalk and root	(Samir et al., 2018)
	*Bacillus subtillis and Trichoderma harzianum*	Reduces infection	(Matić et al., 2013)
In-vitro and in-planta assay	*Microbacterium oleovorans* *Bacillus amyloliquefaciens* *Kluyveromyces sp*	Decreases growth rate of fungi	(Chain EPoCitF et al., 2018)
	*Bacillus megaterium* *Bacillus cereus sensu lato* *Bacillus* sp. *B35*	Decreases fungi severity	(Kamle et al., 2019)
	*Arthrobacter globiformis RC5* *Azotobacter armeniacus RC2*	Inhibits growth rate of pathogens and reduces fungi root colonization	(Alamu et al., 2016)

## 11 Conclusion

The common maize rhizosphere inhabitant and pathogen, *F. verticillioides*, has contributed to various agricultural challenges, including soil contamination. It has led to several recorded disease outbreaks that have killed livestock and contributed to the loss of produced grains (maize). Several techniques have been adopted to eliminate this fungus from plants and fields but to no avail. However, the use of biological agents has been considered an excellent option, which is not only effective but environmentally friendly and safe. *Bacillus spp*, *Enterobacter*, *Pseudomonas, and Microbacterium oleovorans* are proven and efficient microbes that can be utilized to control and inhibit this pathogen. The outbreaks and occurrence of illnesses caused by *F. verticilliodes* and its fumonisin derivative, and the deadly threat they pose, call for a corporate intervention on how this pathogen can be resisted and impeded through the use of biological control agents due to their advantages over synthetic pesticides and fungicides.

## Author contributions

OPO conceptualize the article which was critique by OOB. Both authors agreed on the final version of the manuscript before submission. All authors contributed to the article and approved the submitted version.
