# Corrigendum: *Fusarium verticillioides* of maize plant: Potentials of propitious phytomicrobiome as biocontrol agents

**DOI:** 10.3389/ffunb.2023.1298350

**Published:** 2023-11-29

**Authors:** Oluwadara Pelumi Omotayo, Olubukola Oluranti Babalola

**Affiliations:** Food Security and Safety Focus Area, Faculty of Natural and Agricultural Science, North-West University, Mmabatho, South Africa

**Keywords:** biocontrol agents, *Fusarium verticillioides*, maize pathogen, maize rhizosphere, phytomicrobiome, fumonisin, mycotoxin

In the published article, the reference Figueroa-López et al. (2016) was mistakenly cited in **10 Biocontrol agents**, *10.2 Enterobacter*, paragraph 1. The sentence previously said “The efficiency of rhizospheric *Enterobacter* in controlling *F. verticilliodes* has been confirmed (Figueroa-López et al., 2016).” The correct sentence appears below.

“The efficiency of rhizospheric *Enterobacter* in controlling *F. verticilliodes* especially when used with other bacteria such as *B. amyloliquefaciens* has been confirmed (Pereira et al., 2010).”

In the published article, the reference Figueroa-López et al. (2016) was mistakenly cited in **10 Biocontrol agents**, *10.2 Enterobacter*, paragraph 2. The sentence previously said “According to Figueroa-López (Figueroa-López et al., 2016), *Enterobacter cloacae* has been reported to be an excellent biocontrol agent against root colonization of this Fusarium species in maize (Hinton and Bacon, 1995).” The correct sentence appears below.

“According to Hinton and Bacon (1995), *Enterobacter cloacae* has been reported to be an excellent biocontrol agent against root colonization of this Fusarium species in maize.”

The authors apologize for these errors and state that this does not change the scientific conclusions of the article in any way.

